# The complete chloroplast genome of *Melanosciadium pimpinelloideum* (Apiaceae), an endemic species of China

**DOI:** 10.1080/23802359.2020.1775141

**Published:** 2020-06-09

**Authors:** Jin-Bo Tan

**Affiliations:** Key Laboratory of Bio-Resources and Eco-Environment of Ministry of Education, College of Life Sciences, Sichuan University, Chengdu, P. R. China

**Keywords:** Chloroplast genome, *Melanosciadium pimpinelloideum*, phylogenetic analysis

## Abstract

*Melanosciadium pimpinelloideum* de Boiss. is an endemic species of China. Here, the complete chloroplast genome of *M. pimpinelloideum* is determined. The whole genome size is 164,431 bp in length, composed of a pair of inverted repeats (IRs) of 35,211 bp, a large single-copy region (LSC) of 76,444 bp and a small single-copy region (SSC) of 17,565 bp. The genome contains 136 genes, including 91 protein-coding genes, 37 transfer RNA genes, and 8 ribosomal RNA genes. Phylogenetic analysis with the reported chloroplast genomes showed that *M. pimpinelloideum* and *P. rhomboidea* var. *tenuiloba* formed a well-supported clade, which nested in *Angelica* species. Logically, the result corroborated the previous treatment of *P. rhomboidea* var. *tenuiloba*.

*Melanosciadium pimpinelloideum* de Boiss. (Apiaceae, Apioideae), the type species of this Chinese endemic genus, is mainly occurred in mountainous regions of eastern Chongqing, northwestern Guizhou, western Hubei, southeastern Shannxi and northeastern Yunnan. Previous studies showed that this species nests in *Angelica* sensu lato within Selineae (Zhou et al. 2009; Liao et al. 2013; Wang et al. 2014). *Pimpinella rhomboidea* Diels var. *tenuiloba* Shan & Pu, with reported complete chloroplast genome (Tan and Yu 2018), was treated as a synonym of *M. bipinnatum* (Shan & Pu) Pimenov & Kljuykov based on morphological evidence (Tan et al. 2015). Here, I assembled the complete chloroplast genome of *M. pimpinelloideum*, to explore the systematic placement of this taxon, and to verify the previous treatment of *P. rhomboidea* var. *tenuiloba*.

Fresh leaves of *M. pimpinelloideum* were collected from Pingli County, Shannxi Province, China (32°0′44.2″N, 109°18′4.31″E, 2060 m), and the voucher specimens (*Jin-Bo Tan & Deng-Feng Xie, T0610*) were deposited in the Herbarium of Sichuan University (SZ, accession numbers SZ02002013/4/5/6). Total genomic DNA was extracted using the modified CTAB method (Doyle and Doyle 1987), and achieved the whole genome sequencing on the Illumina Hiseq Platform (Novogene, Beijing, China). I found the published chloroplast genomes of *P. rhomboidea* var. *tenuiloba* was the best reference (GenBank accession no. MG719855) and used it for assembled via program NOVOPlasty (Dierckxsens et al. 2017). The assembled plastid genome was annotated using Geneious v11.0.3 (Kearse et al. 2012) and corrected manually. Finally, the genome map was generated by using the web server OGDRAW (http://ogdraw.mpimp-golm.mpg.de/) (Lohse et al. 2013).

The complete chloroplast genome of *M. pimpinelloideum* (GenBank accession no. MN810920) was 164,431 bp in total length, consisting of a large single copy (LSC, 76,444 bp), a small single copy (SSC, 17,565 bp) and a pair of inverted repeat regions (IRs, 35,211 bp each). 136 genes were detected, including 91 protein-coding genes, 37 transfer RNA genes, and 8 ribosomal RNA genes with overall GC content was 37.5%.

To clarify the phylogenetic relationship between *M. pimpinelloideum* and other Apiaceae taxa, 24 chloroplast genome sequences were achieved from National Center for Biotechnology Information (NCBI, https://www.ncbi.nlm.nih.gov/). After aligned using MAFFT (Katoh et al. 2002), the alignment was used to generate a maximum likelihood (ML) tree via RAxML v8 (Stamatakis 2014) with 1000 bootstrap replicates (Figure 1). The result showed that *M. pimpinelloideum* and of *P. rhomboidea* var. *tenuiloba* formed a well-supported clade with 100% BS value, which nested in *Angelica sensu lato*. The result corroborated the taxonomic treatment of Tan et al. (2015), and the systematic position of *Melanosciadium* in previous studies (Zhou et al. 2009; Liao et al. 2013; Wang et al. 2014). The complete chloroplast genome data provide fundamental information for the recognition and utilization of *M. pimpinelloideum*.

**Figure 1.**
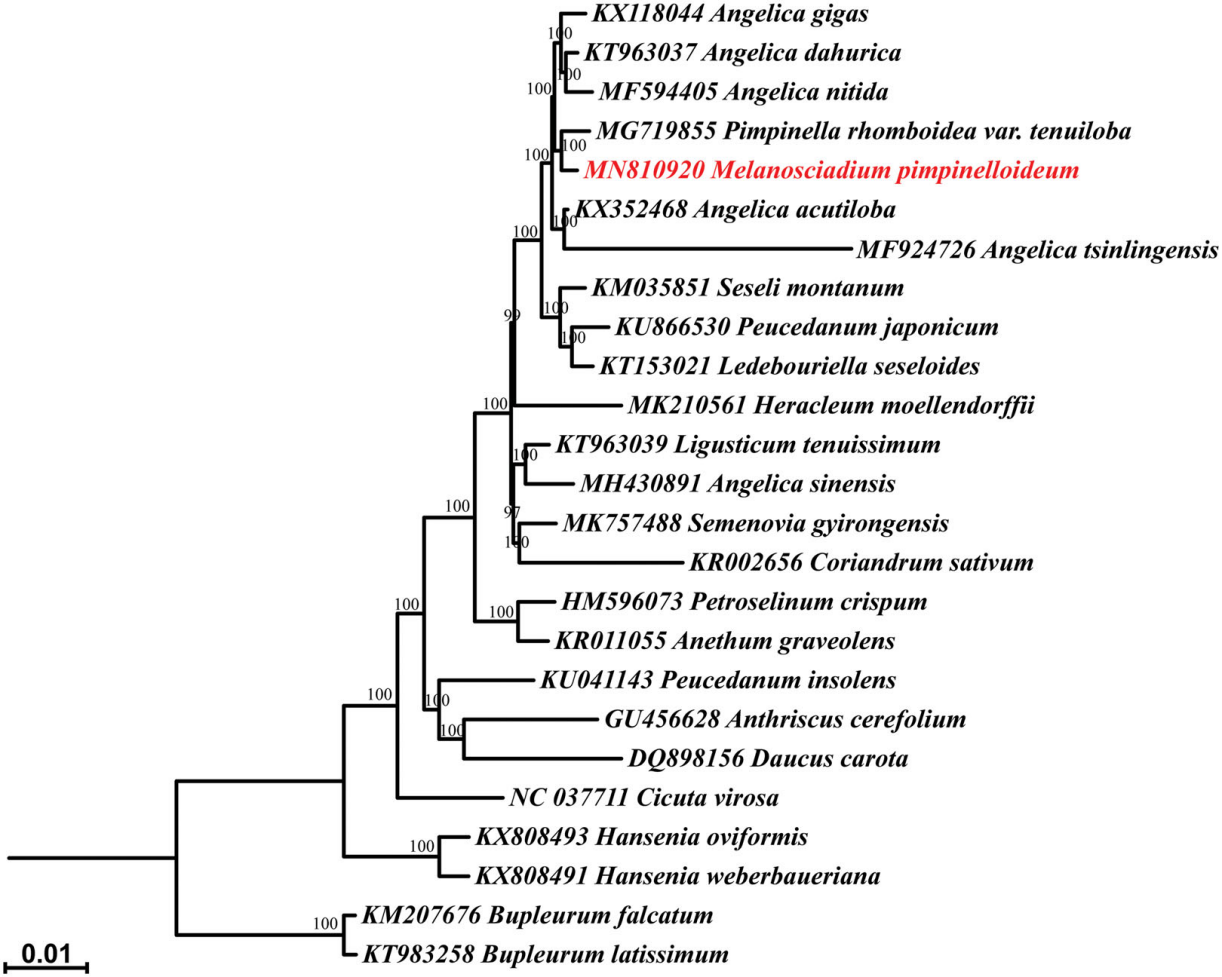
Maximum likelihood (ML) analysis of *Melanosciadium pimpinelloideum* and related species in Apioideae, based on complete chloroplast genome sequences. Numbers on the nodes represent the bootstrap (BS) values from 1000 replicates.

## Data Availability

The data that support the findings of this study are openly available in National Center for Biotechnology Information (NCBI) at https://www.ncbi.nlm.nih.gov/. The GenBank accession number of the complete chloroplast genome of *Melanosciadium pimpinelloideum*, generated in this study, is MN810920.
